# Different prognostic implication of ypTNM stage and pTNM stage for gastric cancer: a propensity score-matched analysis

**DOI:** 10.1186/s12885-019-5283-3

**Published:** 2019-01-16

**Authors:** Ziyu Li, Yinkui Wang, Xiangji Ying, Fei Shan, Zhouqiao Wu, Lianhai Zhang, Shuangxi Li, Yongning Jia, Hui Ren, Jiafu Ji

**Affiliations:** Department of Gastrointestinal Cancer Center Surgery, Key laboratory of Carcinogenesis and Translational Research (Ministry of Education), Peking University Cancer Hospital & Institute, No. 52 Fu-Cheng Road, Hai-Dian District, Beijing, 100142 People’s Republic of China

**Keywords:** Gastric cancer, Neoadjuvant chemotherapy, ypTNM, pTNM, Survival, Propensity score matching

## Abstract

**Background:**

Pathological stage is considered as the best prognosis indicator for gastric cancer. With the increasing use of neoadjuvant chemotherapy (NACT), the latest TNM staging included a new pathological stage of ypTNM for patients with NACT. However, no study has investigated if ypTNM stage has the same prognostic implication as pTNM stage for gastric cancer.

**Methods:**

We retrospectively selected eligible patients within a prospectively maintained database containing all patients treated with gastric cancer in Peking University Cancer Hospital from 2007 to 2015 using overall survival as the outcome. Patients using ypTNM and pTNM were 1:1 matched by propensity scores (PS) calculated from a model containing variables associated with ypTNM use or survival. Overall survival was compared by unconditional Cox regression. Conventional multivariate analysis was conducted to corroborate PS matching results.

**Results:**

1441 patients were included in the analysis with a median follow-up of 37 months (range = 2–106). The matched sample contained 756 patients. After PS matching, patients with specific ypTNM stage were 1.34 (95%CI = 1.05–1.72, *P* = 0.019) times more likely to die than patients with the same pTNM stage. Similar to the results of PS matching, multivariate Cox regression yielded a hazard ratio (HR) of 1.35 (95%CI = 1.09–1.67, *P* = 0.006). Subgroup analysis indicated this survival difference between ypTNM and pTNM stage varied by the specific TNM stage of patients. The HR was 3.44 (95%CI = 1.06–11.18, *P* = 0.040) and 1.28 (95%CI = 1.00–1.62, *P* = 0.048) for patients in stage I and III, respectively; whereas for stage II patients, no significant difference was observed (HR = 1.37, 95%CI = 0.78–2.38, *P* = 0.27).

**Conclusion:**

Gastric cancer patients with specific ypTNM stage had worse prognosis compared to those at the same stage defined by pTNM.

**Electronic supplementary material:**

The online version of this article (10.1186/s12885-019-5283-3) contains supplementary material, which is available to authorized users.

## Background

Gastric cancer is one of the most common cancers worldwide. Due to the high rate of local and systemic recurrence, the survival of gastric cancer patients, especially those in advanced stage, are still not optimistic [1]. During the last decade, preoperative neoadjuvant therapy (NACT) has been recommended as a mean to improve gastric cancer patient prognosis by the National Comprehensive Cancer Network (NCCN) [2], European Society for Medical Oncology (ESMO) [3] and Japanese Gastric Cancer Treatment Guidelines [4].

The Tumor, Node, and Metastasis (TNM) staging system is the most important staging tool for prognosis evaluation of gastric cancer patients. Several revisions have been made to this staging system since its first edition in 1976. The American Joint Commission on Cancer (AJCC) released the latest version (8th) of TNM staging in October 2016. Compared to the 7th edition, one of the highlights is that the new edition takes into account the increasing use of NACT and provides a new pathological TNM staging — post-neoadjuvant therapy TNM (ypTNM) — for staging patients with NACT [5, 6]. Conventionally, pathological stage is considered as the best prognostic indicator for gastric cancer patients; but this conclusion was drawn before the introduction of ypTNM. Moreover, although ypTNM have the same grouping stage as pTNM (i.e. stage I, II, III, IV), a patient’s ypTNM stage might be very different from his/her pTNM stage if he or she had not undergone preoperative NACT, due to the downstaging effect of NACT. Thus, one question comes up: does the new ypTNM stage have the same prognostic implication as the pTNM stage? In clinical practice, should clinicians treat patients with and without NACT the same given a same pathological stage? Given very little previous evidence in this regard, we conducted this retrospective analysis in a prospectively collected cohort to investigate the difference in prognostic value between ypTNM and pTNM stage using propensity score-matching method.

## Method

### Study population

After obtaining approval from Peking University Ethics Committee, a retrospective selection of eligible patients was conducted within a prospectively maintained database consisting of all patients diagnosed and treated with gastric cancer at the Gastrointestinal Cancer Center of Peking University Cancer Hospital & Institute from January 1st 2007 to January 1st 2015. Consent to participate was obtained from every participant. Patient clinicopathological information was stored in this database since their first-time treatment at the hospital. Follow-up was conducted by telephone every three months in the first three years since discharge, every six months in the year 4 and 5, and every six months to one year after five years. If the patient (or contact) could not be reached in three times, the patient was defined as lost-to-follow-up. The last follow-up was carried out in February 2016.

A patient was included in the current study if met the following: 1. Preoperative pathological diagnosis (via biopsy) of gastric adenocarcinoma; 2. Complete clinical and postoperative pathological data; 3. No distant metastasis at diagnosis; 4. Receiving radical D1+/D2 gastrectomy in our center. The exclusion criteria were: 1. Gastrointestinal stromal tumor, lymphoma, neuroendocrine tumor, carcinoid tumors, soft tissue tumors and other non-gastric adenocarcinoma patients diagnosed prior to the treatment; 2. Perioperative death within one month; 3. Receiving chemotherapy for other neoplasms within six months before gastric cancer surgery; 4. Receiving neoadjuvant radiotherapy or targeted therapy before surgery; 5. Remnant stomach cancer; 6. Receiving prophylactic intraperitoneal chemotherapy.

### Exposure, outcome, and confounders

In accordance with the NCCN guideline, NACT was recommended to gastric cancer patients of stage cT2-4NanyM0. Radical D1+/D2 gastrectomy was conducted on all patients. Lymph nodes were identified and dissected by experienced surgeon during the surgery. The metastasis status of resected nodes was determined by one pathologist and reviewed by another afterwards. Because ypTNM was first proposed in 2016, pTNM was used for all patients in the clinical records. We re-grouped the patients who received preoperative NACT using ypTNM staging system according to the 8th AJCC Cancer Staging Manual [6]. However, we used the pTNM stage described in the 7th AJCC Cancer Staging Manual, considering that the 7th edition is still under wide use and the application of the 8th is yet to be validated in clinical settings. Furthermore, the main difference between the 7th and 8th version, which would change our results, is that patients with stage of T1N3b is classified to II in the 7th but to III in the 8th. There was only one patient with such change of staging classification in our study. We created an indicator variable indicating if ypTNM or pTNM was used for the purpose of analysis.

The outcome of interest was overall survival, which was defined as the time interval from the time of the initial therapy to the date of all-cause death or the last follow-up. For patients receiving NACT, the time started from the first cycle chemotherapy after diagnosis with gastric cancer. For patients not receiving NACT, the time of initial therapy was the time receiving radical surgery.

The following demographic characteristic, clinical and pathological information were also extracted from the database to serve as potential confounders or predictors of survival: age, gender, BMI, Eastern Cooperative Oncology Group (ECOG) score, American Society of Anesthesiologists (ASA) score, family history of cancer, operation duration, laparoscopic surgery or not, range of gastric resection, digestive reconstruction, combination with multi-organ resection, blood loss, postoperative hospitalization duration, tumor location, total number of resected lymph node, total number of metastatic lymph nodes, pathological type, differentiation grade, tumor diameter (in long and short axis), and the existence of vascular cancer embolus.

### Statistical analysis

Descriptive statistics are presented as frequencies for categorical variables and mean ± standard deviation for continuous variables. Pearson’s χ2 or Fisher’s exact tests were used to analyze categorical variables. Continuous variables were compared using Student’s T tests if normally distributed or Mann-Whitney U tests if otherwise. Overall survival was calculated using Kaplan-Meier method. We used univariate logistic regression and univariate Cox regression to identify covariates associated with the use of ypTNM and overall survival, respectively.

Propensity score matching is a method widely used to reduce bias due to confounding in non-randomized studies. In this study, we used propensity score matching for the purpose of minimizing confounding as well as another: making the distribution of T stage and N stage comparable between the ypTNM and pTNM group, so to compare the prognostic implication of ypTNM and pTNM staging when the absolute number of the stage was the same. That is, for instance, whether a patient at stage of ypT1a, ypN3a, and M0 had the same overall survival as a patient of pT1a, pN3a, and M0.

We calculated propensity score through a logistic regression model including variables that are significantly associated with the use of ypTNM or overall survival. Quadratic terms of continuous variables were added to the propensity score model to account for non-linearity if appropriate. Considering the propensity score was not normally distributed, we matched the sample on the logit of the calculated propensity score. The greedy nearest neighbor matching algorithm without replacement was used at a 1:1 ratio. A caliper size of 0.2 of the standard deviation of the logit of the propensity score was utilized, as such a caliper size had the most superior performance on reducing bias among the commonly used sizes in current clinical research according to prior Monte Carlo simulations [7–9]. Mann-Whitney U and Pearson’s χ2 tests were used to check if the clinicopathological characteristics between the two groups were balanced. Following propensity score-matching, overall survival between matched ypTNM and pTNM patients was examined by unconditional Cox regression.

Conventional multivariate analysis was next used to verify the results from propensity score matching. All variables in the propensity score calculation model were included in this multivariate Cox model. This method could likewise achieve the two purposes of propensity score matching mentioned above. Furthermore, a subgroup analysis by pathological stages (i.e. I, II, III) was performed using the multivariate models to assess if the difference of prognostic implication of ypTNM and pTNM differed by stage.

The assumption of proportional hazards were examined using Schoenfeld residual. All data analyses was performed with Stata software version 14 (College Station, TX: StataCorp LP) and Rstudio version 1.1.419 (RStudio, Inc., Boston, MA) with a two-sided *p* < 0.05 defined as statistically significant.

## Results

### Patient characteristics of the unmatched cohort

**Fig. 1:**
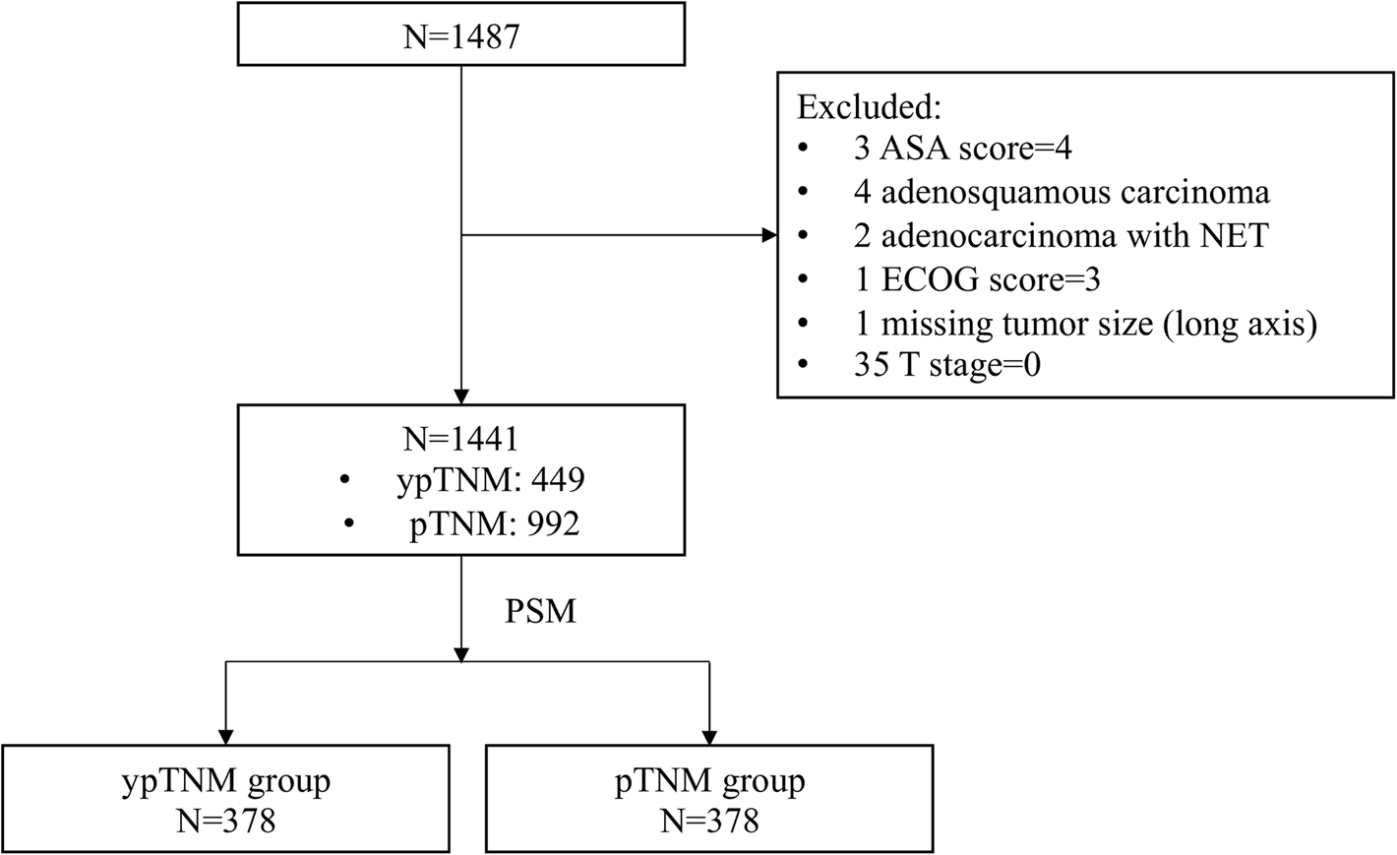
Study design and exclusion criteria

1487 eligible patients were included in this study, of which 46 were excluded, leaving a sample of 1441 for analysis (Fig. 1). Table 1 shows the clinicopathological characteristics of the unmatched sample. The average age was 59.2 ± 11.4 years, of which 397 (27.55%) were female. More than half of the patients had tumor located at low stomach (54.41%). Most patients had adenocarcinoma (75.57%). Most tumors were at moderate differentiation grade (46.22%), ypT or pT stage of T4a (47.95%), and ypN or pN stage of N0 (42.05%). Open gastrectomy was performed on 87.09% of the sample. The median follow-up for all patients, the ypTNM group, and the pTNM group was 37 (range = 2–106), 36 (range = 3–106), and 37 months (range = 2–106), respectively. The 3-year overall survival for all patients, the ypTNM group, and the pTNM group was 72.12, 62.40, and 76.92%, respectively.

**Table 1:** Baseline clinical and pathological characteristics of patients using ypTNM stage or pTNM stage in Peking University Cancer Hospital & Institute, 2007–2015 (*n* = 1441)

Characteristics	Total (*N* = 1441)	ypTNM (*N* = 449)	pTNM (*N* = 992)	*P* value^*^
Age (μ ± sd)	59.2 ± 11.4	59.3 ± 10.2	59.1 ± 11.9	0.69
Female	397 (27.55%)	102 (22.72%)	295 (29.74%)	0.006
Family history of cancer (yes)	328 (22.76%)	81 (18.04%)	247 (24.9%)	0.004
BMI (μ ± sd, kg/m2)	23.5 ± 3.4	23.4 ± 3.3	23.5 ± 3.5	0.71
ECOG				0.08
0	928 (64.4%)	290 (64.59%)	638 (64.31%)	
1	419 (29.08%)	139 (30.96%)	280 (28.23%)	
2	94 (6.52%)	20 (4.45%)	74 (7.46%)	
Tumor location				< 0.001
Upper	393 (27.27%)	166 (36.97%)	227 (22.88%)	
Middle	212 (14.71%)	59 (13.14%)	153 (15.42%)	
Low	784 (54.41%)	195 (43.43%)	589 (59.38%)	
Whole	52 (3.61%)	29 (6.46%)	23 (2.32%)	
Short diameter (μ ± sd, in cm)	2.9 ± 2.1	3.1 ± 2.4	2.9 ± 2.0	0.21
Long diameter (μ ± sd, in cm)	4.2 ± 2.8	4.5 ± 3.1	4.0 ± 2.6	0.035
Pathological type				< 0.001
Adenocarcinoma	1089 (75.57%)	356 (79.29%)	733 (73.89%)	
Signet ring cell carcinoma^a^	290 (20.12%)	67 (14.92%)	223 (22.48%)	
Mucinous adenocarcinoma^b^	62 (4.3%)	26 (5.79%)	36 (3.63%)	
Differentiation grade				0.029
Well	159 (11.03%)	36 (8.02%)	123 (12.4%)	
Moderate	666 (46.22%)	206 (45.88%)	460 (46.37%)	
Poor	616 (42.75%)	207 (46.1%)	409 (41.23%)	
T stage				< 0.001
T1	296 (20.54%)	35 (7.8%)	261 (26.31%)	
T2	238 (16.52%)	68 (15.14%)	170 (17.14%)	
T3	216 (14.99%)	66 (14.7%)	150 (15.12%)	
T4	691 (47.95%)	280 (62.36%)	411 (41.43%)	
N stage				0.002
N0	606 (42.05%)	157 (34.97%)	449 (45.26%)	
N1	249 (17.28%)	94 (20.94%)	155 (15.63%)	
N2	234 (16.24%)	84 (18.71%)	150 (15.12%)	
N3a	224 (15.54%)	67 (14.92%)	157 (15.83%)	
N3b	128 (8.88%)	47 (10.47%)	81 (8.17%)	
TNM stage				< 0.001
IA	261 (18.11%)	25 (5.57%)	236 (23.79%)	
IB	159 (11.03%)	43 (9.58%)	116 (11.69%)	
IIA	110 (7.63%)	30 (6.68%)	80 (8.06%)	
IIB	227 (15.75%)	102 (22.72%)	125 (12.6%)	
IIIA	185 (12.84%)	76 (16.93%)	109 (10.99%)	
IIIB	237 (16.45%)	85 (18.93%)	152 (15.32%)	
IIIC	262 (18.18%)	88 (19.6%)	174 (17.54%)	
Vascular cancer embolus (yes)	876 (60.79%)	291 (64.81%)	585 (58.97%)	0.035
Hospital stay (μ ± sd, in days)	14.2 ± 13.8	14.6 ± 16.8	13.9 ± 11.9	0.11
Operative time (μ ± sd, in min)	206.2 ± 64.4	212.1 ± 63.6	203.6 ± 64.7	0.003
Blood loss (μ ± sd, in ml)	150.6 ± 210.7	175.2 ± 324.6	139.5 ± 128.4	< 0.001
Gastrectomy type				< 0.001
Laparoscopic^c^	186 (12.91%)	24 (5.35%)	162 (16.33%)	
Open	1255 (87.09%)	425 (94.65%)	830 (83.67%)	
Resection range				< 0.001
Total	496 (34.42%)	210 (46.77%)	286 (28.83%)	
Distal	785 (54.48%)	171 (38.08%)	614 (61.9%)	
Proximal	141 (9.78%)	55 (12.25%)	86 (8.67%)	
Thoraticabdominal joint	19 (1.32%)	13 (2.9%)	6 (0.6%)	
Multi-organ excision (yes)	80 (5.55%)	36 (8.02%)	44 (4.44%)	0.006
Reconstruction approach				< 0.001
Billroth II	342 (23.73%)	77 (17.15%)	265 (26.71%)	
Billroth I	412 (28.59%)	88 (19.6%)	324 (32.66%)	
Roux-en-Y	369 (25.61%)	138 (30.73%)	231 (23.29%)	
Jejunal interposition	165 (11.45%)	83 (18.49%)	82 (8.27%)	
Other	153 (10.62%)	63 (14.03%)	90 (9.07%)	
ASA				0.21
1	205 (14.23%)	60 (13.36%)	145 (14.62%)	
2	969 (67.24%)	316 (70.38%)	653 (65.83%)	
3	267 (18.53%)	73 (16.26%)	194 (19.56%)	
N of lymph nodes dissected (μ ± *sd*)	31.0 ± 12.0	32.0 ± 12.2	30.5 ± 11.9	0.06
N of lymph nodes metastasis (μ ± *sd*)	4.7 ± 7.5	5.2 ± 8.0	4.4 ± 7.2	0.004

^a^include adenocarcinoma with signet ring

^b^include adenocarcinoma with mucinous adenocarcinoma, mucinous adenocarcinoma with signet ring

^c^include total laparoscopic and laparoscopic-assisted gastrectomy

^*^Mann-Whitney U test was used for all continuous variables

### Characteristics of the propensity score-matched sample

To better control for confounding and achieve comparable distribution of T and N stage between the two groups, patients were matched 1:1 based upon factors associated with the likelihood of using ypTNM staging or survival hazard in the unmatched cohort (Additional file 1: Table S1). The propensity score-matched sample were comprised 756 patients (378 in each group). Table 2 displays the covariate differences between the ypTNM and pTNM group after matching. All previously observed covariate imbalances between the two groups were no longer significant after matching. Moreover, matching balanced the distribution of factors associated with hazard of all mortality. As such, matching was considered effective. The clinicopathological characteristics of the matched sample were somewhat similar to the unmatched cohort. The majority of patients had locally advanced gastric cancer (stage III [53.84%], node positivity [64.29%]).

**Table 2:** Comparison of Clinicopathological Variables between ypTNM group and pTNM (*n* = 756) after Propensity Score-matching Using the Greedy Nearest Neighbor Algorithm without Replacement (Caliper Width 0.2 × Standard Deviation of Logit Propensity Score])

Characteristics	ypTNM (*N* = 378)	pTNM (N = 378)	P value^*^
Age (μ ± sd)	59.2 ± 10.5	59.3 ± 10.7	0.82
Female	97 (25.66%)	88 (23.28%)	0.45
Family history of cancer (yes)	74 (19.58%)	71 (18.78%)	0.78
BMI (μ ± sd, kg/m2)	23.5 ± 3.3	23.5 ± 3.6	0.96
ECOG			0.81
0	249 (65.87%)	257 (67.99%)	
1	111 (29.37%)	105 (27.78%)	
2	18 (4.76%)	16 (4.23%)	
Tumor location			0.99
Upper	120 (31.75%)	131 (34.66%)	
Middle	53 (14.02%)	53 (14.02%)	
Low	185 (48.94%)	178 (47.09%)	
Whole	20 (5.29%)	16 (4.23%)	
Short diameter (μ ± sd, in cm)	3.1 ± 2.4	3.1 ± 2.0	0.59
Long diameter (μ ± sd, in cm)	4.5 ± 3.1	4.4 ± 2.8	0.72
Pathological type			0.97
Adenocarcinoma	299 (79.1%)	298 (78.84%)	
Signet ring cell carcinoma^1^	62 (16.4%)	64 (16.93%)	
Mucinous adenocarcinoma^2^	17 (4.5%)	16 (4.23%)	
Differentiation grade			0.97
Well	31 (8.2%)	30 (7.94%)	
Moderate	175 (46.3%)	174 (46.03%)	
Poor	172 (45.5%)	174 (46.03%)	
T stage			0.98
T1	35 (9.26%)	37 (9.79%)	
T2	65 (17.2%)	66 (17.46%)	
T3	59 (15.61%)	55 (14.55%)	
T4	219 (57.94%)	220 (58.2%)	
N stage			0.99
N0	135 (35.71%)	139 (36.77%)	
N1	78 (20.63%)	78 (20.63%)	
N2	63 (16.67%)	58 (15.34%)	
N3a	60 (15.87%)	61 (16.14%)	
N3b	42 (11.11%)	42 (11.11%)	
TNM stage			0.48
IA	25 (6.61%)	33 (8.73%)	
IB	42 (11.11%)	48 (12.70%)	
IIA	27 (7.14%)	28 (7.41%)	
IIB	83 (21.96%)	63 (16.67%)	
IIIA	63 (16.67%)	55 (14.55%)	
IIIB	60 (15.87%)	69 (18.25%)	
IIIC	78 (20.63%)	82 (21.69%)	
Vascular cancer embolus (yes)	234 (61.9%)	239 (63.23%)	0.71
Hospital stay (μ ± sd, in days)	14.2 ± 12.3	14.2 ± 11.8	0.39
Operative time (μ ± sd, in min)	206.5 ± 58.5	208.8 ± 58.5	0.59
Blood loss (μ ± sd, in ml)	153.6 ± 120.9	153.8 ± 153.4	0.22
Gastrectomy type			0.76
Laparoscopic^3^	24 (6.35%)	22 (5.82%)	
Open	354 (93.65%)	356 (94.18%)	
Resection range			0.85
Total	159 (42.06%)	165 (43.65%)	
Distal	167 (44.18%)	164 (43.39%)	
Proximal	48 (12.7%)	43 (11.38%)	
Thoraticabdominal joint	4 (1.06%)	6 (1.59%)	
Multi-organ excision (yes)	19 (5.03%)	23 (6.08%)	0.53
Reconstruction approach			0.77
Billroth II	76 (20.11%)	85 (22.49%)	
Billroth I	86 (22.75%)	73 (19.31%)	
Roux-en-Y	115 (30.42%)	119 (31.48%)	
Jejunal interposition	52 (13.76%)	55 (14.55%)	
Other	49 (12.96%)	46 (12.17%)	
ASA			0.21
1	57 (15.08%)	41 (10.85%)	
2	254 (67.2%)	270 (71.43%)	
3	67 (17.72%)	67 (17.72%)	
N of lymph nodes dissected (μ ± *sd*)	32.4 ± 11.3	31.7 ± 12.2	0.22
N of lymph nodes metastasis (μ ± *sd*)	5.4 ± 8.5	5.2 ± 8.0	0.68

^1^include adenocarcinoma with signet ring

^2^include adenocarcinoma with mucinous adenocarcinoma, mucinous adenocarcinoma with signet ring

^3^include total laparoscopic and laparoscopic-assisted gastrectomy

^*^Mann-Whitney U test was used for all continuous variables

### Prediction implication of ypTNM versus pTNM on survival

**Fig. 2:**
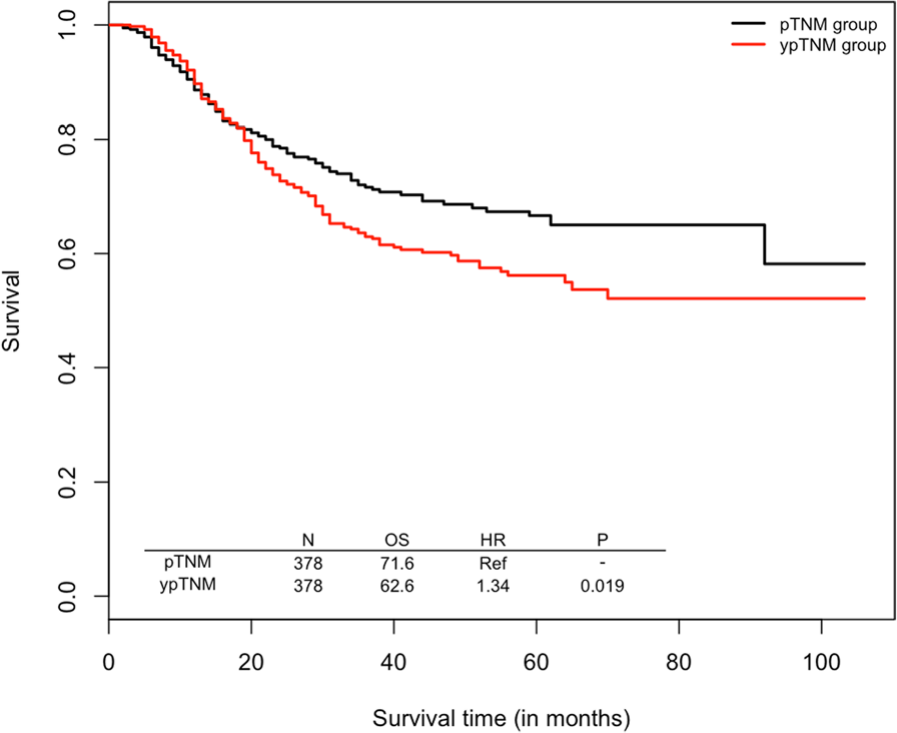
Comparative prognosis implication of ypTNM vs yTNM on overall survival in propensity-matched cohorts of patients with gastric cancer. *OS is three-year overall survival

Unconditioned Cox regression on the matched sample yielded a hazard ratio of 1.34 comparing ypTNM group to pTNM group (95%CI = 1.05–1.72, *P* = 0.019; Fig. 2). We calculated Harrell’s c-index to compare the prognostic prediction ability of ypTNM staging and pTNM staging. The model with ypTNM as the staging system achieved a c-statistic of 0.67, which appeared to be smaller than that of the model with pTNM (0.69).

**Fig. 3:**
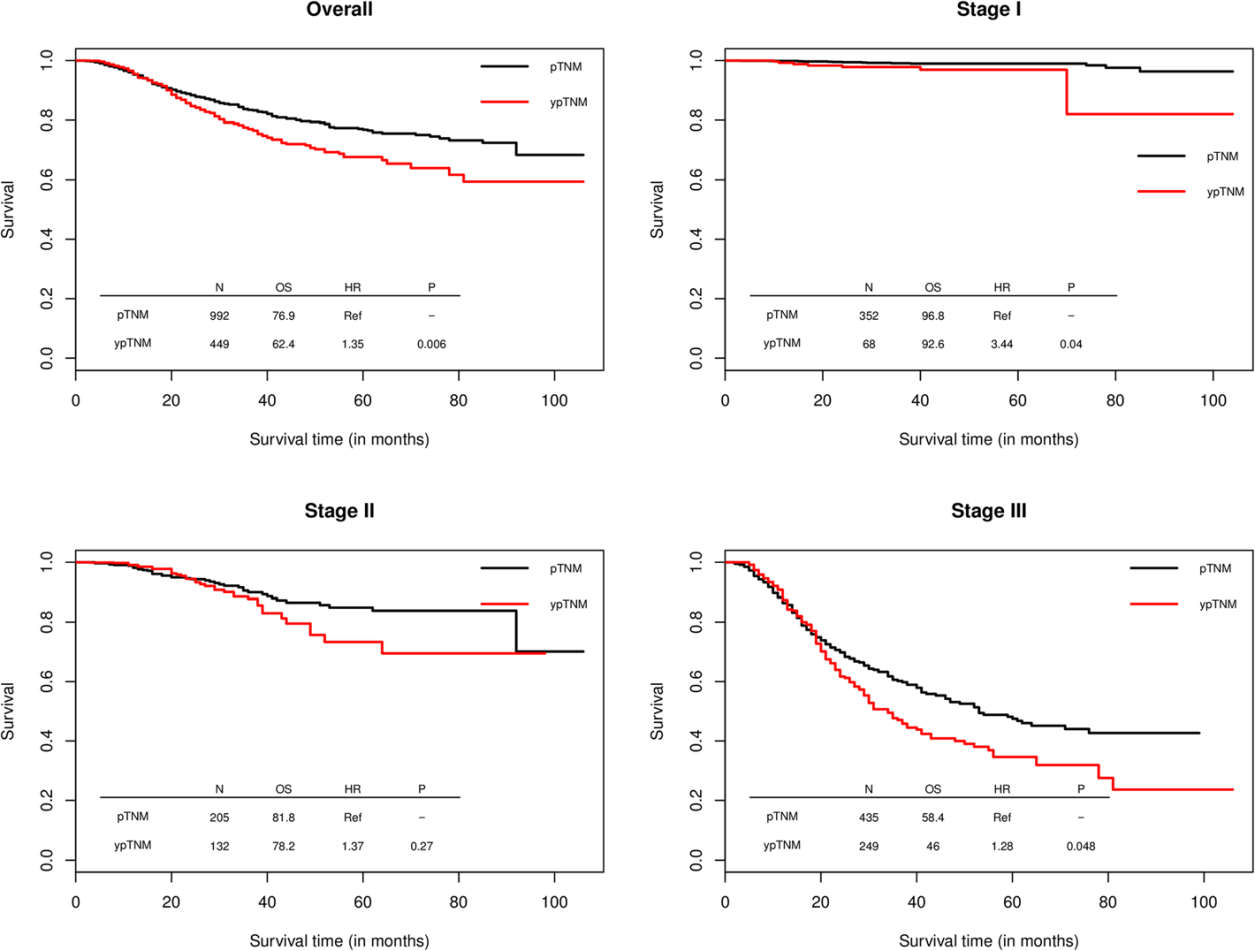
Adjusted comparative prognosis implication of ypTNM vs yTNM on overall survival in unmatched cohorts of patients with gastric cancer, stratified by TNM stage. *OS is three-year overall survival

Conventional multivariate analysis was used to corroborate the results from propensity score matching. After adjusting for all variables included in the propensity score matching model (Additional file 1: Table S1), patients with specific ypTNM stage were 1.35 times more likely to die than patients with the same pTNM stage (95%CI = 1.09–1.67, *P* = 0.006). Further subgroup analysis using multivariate Cox models indicated this survival difference between ypTNM and pTNM group varied by TNM stage (Fig. 3). For patients in stage I and III, the adjusted hazard ratio was 3.44 (95%CI = 1.06–11.18, *P* = 0.040) and 1.28 (95%CI = 1.00–1.62, *P* = 0.048) comparing ypTNM to pTNM, respectively; whereas for patients in stage II, no significant difference was observed in terms of the prediction implication of ypTNM and pTNM stage on survival (adjusted HR = 1.37, 95%CI = 0.78–2.38, *P* = 0.27). The Kaplan-Meier Curves by detailed TNM staging (e.g. Ia, Ib, etc) are shown in Additional file 1: Figure S1.

### Overall survival for pT0 or ypT0

4 pT0 and 31 ypT0 patients were not included in the analysis above due to the small group size. These 4 patients had one-bite gastric cancer. 28 of the ypT0 patients were with pathological complete response and the remaining 3 had ypT0N1. The 3-year overall survival for the pT0 and ypT0 patients was 100 and 96.3%, respectively. The 5-year overall survival for the pT0 and ypT0 patients was 100 and 89.9%, respectively.

## Discussion

The latest edition of AJCC Cancer Staging Manual presented a comprehensive TNM staging system for clinicians to use under different situations, including clinical TNM (cTNM), pathological TNM (pTNM) and post-neoadjuvant therapy TNM (ypTNM). cTNM stage is considered of great value when determining clinical intervention, but its significance in survival prediction is limited compared to traditional pTNM. Furthermore, several studies have compared the prognostic value of cTNM to ypTNM on gastric cancer patients [10–12], but none focused on the comparison between ypTNM and pTNM. The current study investigated whether the same pathological stage defined by ypTNM and pTNM predicted similar survival in patients with gastric cancer. To our best knowledge, this study is the first of its kind.

We found patients with specific ypTNM stage had worse prognosis compared to those at the same pathological stage defined by pTNM. This result is reasonable: if two patients shared a same pathological stage defined by ypTNM and pTNM, due to the downstaging effect of NACT, the patient with ypTNM stage would have a higher pTNM stage if had not received NACT, making it logical that this patient’s prognosis was worse.

Our subgroup analysis further indicated that this difference on prognostic value of ypTNM versus pTNM differed by pathological stage. Interestingly, the magnitude of effect comparing ypTNM to pTNM was largest in stage I, followed by stage III, and was not significant in stage II. Potential explanations for this phenomenon are as follow. Because patients in pStage I did not normally use NACT, the majority of patients in ypStage I should be those who responded well to NACT and thus downstaged by NACT from stage II and III. [13] These patients would therefore have much worse prognosis than those in pTNM stage I because they would have a worse pTNM stage have they had one. As patients with distant metastasis (i.e. stage IV) were excluded from our analysis, patients in ypStage III were those who did not respond to NACT and remained in stage III after NACT. These patients had worse prognosis compared to patients with pStage III because they were insensitive to chemotherapy; but the magnitude of difference was not as great as that for those in ypStage I. Patients in ypStage II might consist of two groups: one group were those downstaged by NACT from stage III, and the remainder were patients who did not respond well to NACT; the mixture of these two groups resulted in a non-significant hazard ratio.

To verify these explanations, we would need information on the counterfactual pTNM stage of the patients who underwent NACT, which is unrealistic. One alternative, if not the only one, is to use cTNM as the surrogate of pTNM. However, when doing so, one should consider the magnitude of correlation between cTNM and pTNM stage. Previous studies have found less than 60–70% cTNM stages were in conformity with pTNM stage [14, 15]. According to a more recent study conducted by the Shizuoka Cancer Center in Japan, this inconformity varied by stage [16]. Nonetheless, despite the imperfect correlation of cTNM with pTNM, it would be still worthwhile to use cTNM as an alternative considering information on pTNM is unavailable for patients with NACT.

Our results not only suggest a different prognostic value of ypTNM and pTNM, but also indicate that the treatment for patients with specific ypTNM stage should be more intensified than that for patients with the same pathological stage defined by pTNM. This difference on treatment should also vary by TNM stage according to our subgroup analysis. For instance, as recommended by the NCCN guideline (5th edition, 2017), postoperative follow-up is sufficient for patients in pStage I, whereas adjuvant chemotherapy is needed for patients in ypStage I [17]. However, the guideline does not provide evidence for this recommendation and our findings offer one theoretical evidence base.

Furthermore, we want to clarify that our results do not indicate any worse survival due to NACT. What we found was given a same pathological stage the patients with NACT had a worse survival than the patients without it. Such phenomenon are mainly due to the down stage effect of NACT [18, 19]. If removing the premise of same pathological stage, multiple previous studies have found a favorable association between NACT and overall survival for gastric cancer patients [13, 20, 21].

There are two main limitations embedded in our study. Firstly, as previously mentioned, because information on cTNM was unavailable for this study, we cannot confirm the explanation of our results. However, this does not affect the conclusion of current study, as we only considered the prognostic value of pTNM and ypTNM stage. Secondly, Borrmann type has been considered as an important prognostic factor of gastric cancer and is one of the indications of NACT in countries such as Japan. Due to the lack of information, this factor was not included in the propensity score or the conventional multivariate analysis. However, given that NACT indications in China do not contain patient Borrmann type, omitting this factor will not introduce bias to the HR estimation.

To sum up, the prognostic implication of ypTNM is different from that of pTNM stage. In particular, patients with specific ypTNM stage had worse prognosis compared to those at the same pathological stage defined by pTNM. Such difference differed by TNM stage. Our findings should be taken into account when predicting the prognosis of and deciding postoperative treatment for patients with NACT using ypTNM.

## Additional file

Additional file 1: **Table S1.** Univariate analysis of clinical and pathological characteristics associated with using ypTNM stage and patient survival. **Figure S1.** Adjusted comparative prognosis implication of ypTNM vs yTNM on overall survival in unmatched cohorts of patients with gastric cancer, stratified by detailed TNM stage (DOCX 1083 kb)

## Abbreviations

AJCC: American Joint Commission on Cancer
ESMO: European Society for Medical Oncology
HR: Hazard ratio
NACT: Neoadjuvant chemotherapy
NCCN: National Comprehensive Cancer Network
TNM: Tumor, node, metastasis
