# Supplementary material for: Different prognostic implication of ypTNM stage and pTNM stage for gastric cancer: a propensity score-matched analysis
Source: BMC Cancer. 2019 Jan 16;19:80. doi: 10.1186/s12885-019-5283-3 (PMC6335703; doi:10.1186/s12885-019-5283-3)
Supplement: Supplementary file 1 — Table S1. Univariate analysis of clinical and pathological characteristics associated with using ypTNM stage and patient survival. Figure S1. Adjusted comparative prognosis implication of ypTNM vs yTNM on overall survival in unmatched cohorts of patients with gastric cancer, stratified by detailed TNM stage (DOCX 1083 kb) [file 12885_2019_5283_MOESM1_ESM.docx]

Appendix

Table 1. Univariate analysis of clinical and pathological characteristics associated with using ypTNM stage and patient survival

| Characteristics | Using ypTNM | | Overall Survival | |
| --- | --- | --- | --- | --- |
|  | OR (95%CI) | P | HR (95%CI) | P |
| Age | 1.00 (0.99, 1.01) | 0.28 | 1.02 (1.02, 1.03) | <0.001 |
| Female | 0.69 (0.54, 0.90) | 0.006 | 0.82 (0.66, 1.03) | 0.08 |
| Family history of cancer | 0.66 (0.50, 0.88) | 0.004 | 0.90 (0.72, 1.13) | 0.35 |
| BMI | 0.99 (0.96, 1.02) | 0.57 | 0.94 (0.92, 0.97) | <0.001 |
| ECOG |  | 0.08 |  | <0.001 |
| 0 | Referent |  | Referent |  |
| 1 | 1.09 (0.85, 1.39) |  | 0.97 (0.79, 1.20) |  |
| 2 | 0.59 (0.36, 0.99) |  | 2.03 (1.50, 2.74) |  |
| Tumor location |  | <0.001 |  | <0.001 |
| Upper | Referent |  | Referent |  |
| Middle | 0.53 (0.37, 0.76) |  | 0.85 (0.63, 1.15) |  |
| Low | 0.45 (0.35, 0.59) |  | 0.65 (0.53, 0.81) |  |
| Whole | 1.72 (0.96, 3.09) |  | 4.16 (2.96, 5.86) |  |
| Short diameter | 1.05 (0.997, 1.105) | 0.07 | 1.20 (1.17, 1.23) | <0.001 |
| Long diameter | 1.05 (1.01, 1.09) | 0.009 | 1.00 (1.00, 1.01) | 0.004 |
| Pathological type |  | 0.001 |  | 0.039 |
| Adenocarcinoma | Referent |  | Referent |  |
| Signet ring cell carcinoma^1^ | 0.62 (0.46, 0.84) |  | 1.31 (1.05, 1.64) |  |
| Mucinous adenocarcinoma^2^ | 1.49 (0.88, 2.50) |  | 1.33 (0.89, 1.99) |  |
| Differentiation grade |  | 0.025 |  | 0.041 |
| Well | Referent |  | Referent |  |
| Moderate | 1.53 (1.02, 2.30) |  | 0.70 (0.53, 0.92) |  |
| Poor | 1.73 (1.15, 2.60) |  | 0.80 (0.60, 1.05) |  |
| T stage |  | <0.001 |  | <0.001 |
| T1 | Referent |  | Referent |  |
| T2 | 2.98 (1.90, 4.68) |  | 5.00 (2.58, 9.72) |  |
| T3 | 3.28 (2.08, 5.18) |  | 10.49 (5.51, 19.96) |  |
| T4 | 5.08 (3.56, 7.46) |  | 15.80 (8.67, 28.83) |  |
| N stage |  | 0.002 |  | <0.001 |
| N0 | Referent |  | Referent |  |
| N1 | 1.73 (1.27, 2.37) |  | 2.23 (1.60, 3.12) |  |
| N2 | 1.60 (1.16, 2.21) |  | 4.28 (3.18, 5.77) |  |
| N3a | 1.22 (0.87, 1.71) |  | 5.30 (3.94, 7.13) |  |
| N3b | 1.66 (1.11, 2.48) |  | 12.34 (9.10, 16.75) |  |
| TNM stage |  | <0.001 |  | <0.001 |
| IA | Referent |  | Referent |  |
| IB | 3.50 (2.04, 6.01) |  | 2.72 (1.13, 6.55) |  |
| IIA | 3.54 (1.97, 6.37) |  | 4.37 (1.81, 10.55) |  |
| IIB | 7.70 (4.73, 12.55) |  | 10.22 (4.90, 21.30) |  |
| IIIA | 6.58 (3.97, 10.91) |  | 13.02 (6.24, 27.15) |  |
| IIIB | 5.28 (3.23, 8.62) |  | 19.68 (9.60, 40.37) |  |
| IIIC | 4.77 (2.94, 7.56) |  | 34.24 (16.84, 69.58) |  |
| Vascular cancer embolus | 1.28 (1.02, 1.61) | 0.035 | 0.35 (0.29, 0.42) | <0.001 |
| Hospital stay | 1.00 (0.99, 1.01) | 0.24 | 1.007 (1.003, 1.01) | 0.004 |
| Operative time | 1.00 (1.000, 1.004) | 0.021 | 1.00 (1.003, 1.005) | <0.001 |
| Blood loss | 1.001 (1.000, 1.002) | 0.006 | 1.00 (1.000, 1.000) | 0.039 |
| Gastrectomy type |  | <0.001 |  | <0.001 |
| Laparoscopic^3^ | Referent |  | Referent |  |
| Open | 3.46 (2.22, 5.39) |  | 2.60 (1.71, 3.96) |  |
| Resection range |  | <0.001 |  | <0.001 |
| Total | Referent |  | Referent |  |
| Distal | 0.38 (0.30, 0.49) |  | 0.41 (0.33, 0.50) |  |
| Proximal | 0.87 (0.59, 1.28) |  | 0.48 (0.34, 0.66) |  |
| Thoratic-abdominal joint | 2.95 (1.10, 7.89) |  | 2.55 (1.51, 4.30) |  |
| Multi-organ excision | 1.88 (1.19, 2.96) | 0.008 | 1.95 (1.42, 2.69) | <0.001 |
| Reconstruction approach |  | <0.001 |  | <0.001 |
| Billroth II | Referent |  | Referent |  |
| Billroth I | 0.93 (0.66, 1.32) |  | 0.91 (0.66, 1.24) |  |
| Roux-en-Y | 2.06 (1.48, 2.86) |  | 2.34 (1.74, 3.14) |  |
| Jejunal interposition | 3.48 (2.34, 5.18) |  | 2.78 (2.03, 3.83) |  |
| Other | 2.41 (1.60, 3.63) |  | 1.34 (0.92, 1.93) |  |
| ASA |  | 0.21 |  | 0.035 |
| 1 | Referent |  | Referent |  |
| 2 | 1.17 (0.84, 1.63) |  | 1.11 (0.85, 1.45) |  |
| 3 | 0.91 (0.61, 1.36) |  | 1.46 (1.07, 2.01) |  |
| N of lymph nodes dissected | 1.01 (1.00, 1.02) | 0.034 | 1.02 (1.01, 1.02) | <0.001 |
| N of lymph nodes metastasis | 1.01 (0.99, 1.03) | 0.08 | 1.07 (1.06, 1.08) | <0.001 |

^1^ include adenocarcinoma with signet ring

^2^ include adenocarcinoma with mucinous adenocarcinoma, mucinous adenocarcinoma with signet ring

^3^ include total laparoscopic and laparoscopic-assisted gastrectomy

Figure 1. Adjusted comparative prognosis implication of ypTNM vs yTNM on overall survival in unmatched cohorts of patients with gastric cancer, stratified by detailed TNM stage


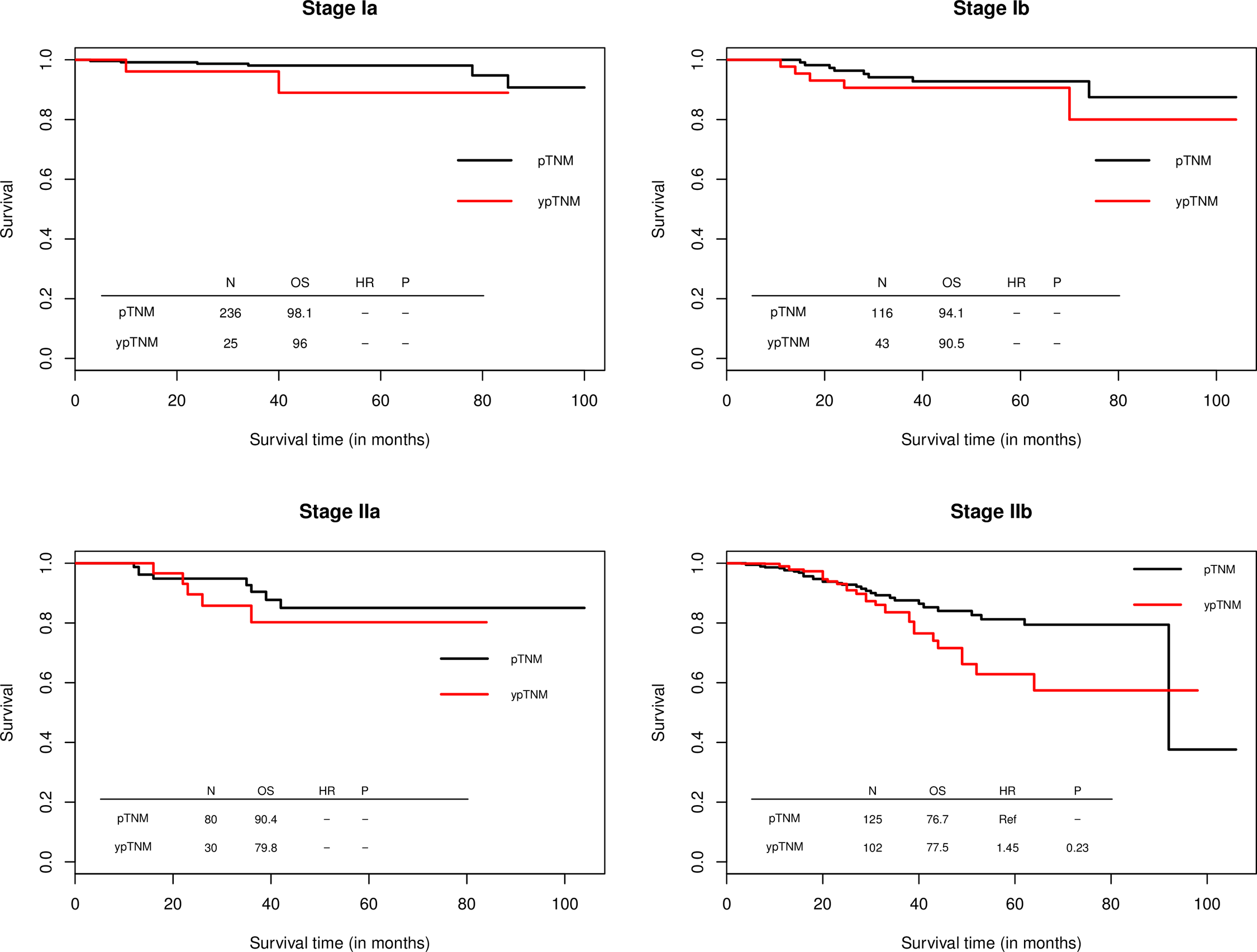


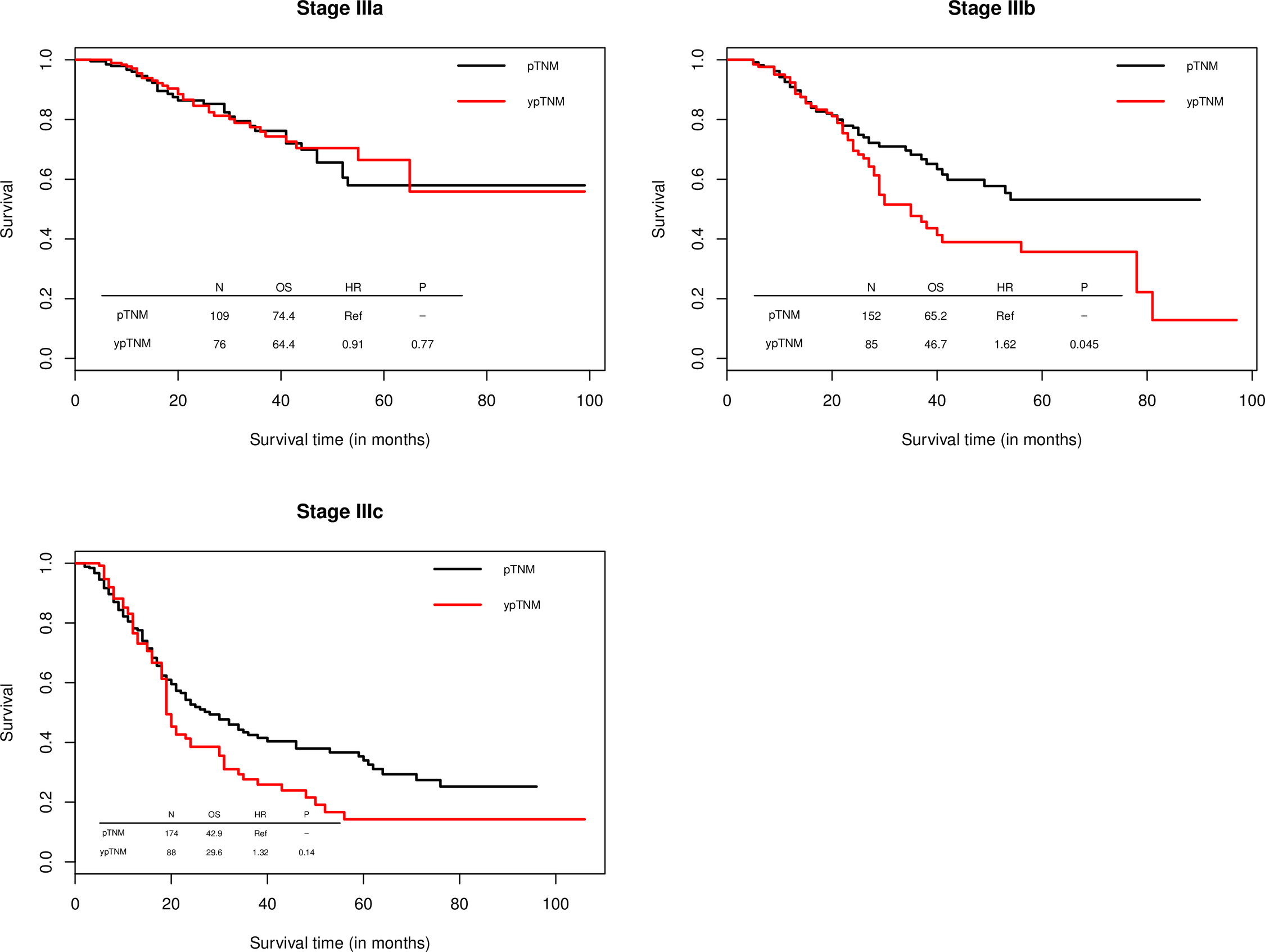


*OS is three-year overall survival. For stage Ia, Ib, and IIa, the Cox model was unable to converge and therefore hazard ratio and p-value were not estimable; The Kaplan-Meier curves for stage Ia, Ib, and IIa were therefore unadjusted.
